# Psychological Distress and Cardiovascular Disease: The Circulatory Risk in Communities Study (CIRCS)

**DOI:** 10.2188/jea.JE20100011

**Published:** 2010-05-05

**Authors:** Tetsuya Ohira

**Affiliations:** Department of Social and Environmental Medicine, Graduate School of Medicine, Osaka University, Suita, Japan; Osaka Medical Center for Health Science and Promotion, Osaka, Japan

**Keywords:** anger, cardiovascular disease, depression, population-based, tension-anxiety

## Abstract

Although a number of epidemiological studies have reported that psychological factors are associated with increased risk of cardiovascular morbidity and mortality, the relevant epidemiological data are mostly limited to Western populations. The present study sought to examine associations of depressive symptoms, anger expression, and tension with the incidence of cardiovascular disease in the Circulatory Risk in Communities Study (CIRCS). Depressive symptoms were measured in 901 men and women by using the Zung Self-Rating Depression Scale (SDS); anger expression and tension were measured in 6292 men and women by using the Anger Expression Scale and Framingham Tension Scale. As compared with the participants with SDS scores in the lowest tertile, those with scores in the highest tertile had twice the age- and sex-adjusted hazard ratio of total stroke. However, this excess risk was present only for ischemic stroke. Participants in the highest tertile also had a 7-fold adjusted hazard ratio of coronary heart disease, as compared with those in the lowest tertile. These associations were virtually unchanged after further adjustment for covariates. Men with “anger-in” scores in the highest tertile had a 1.5-fold age-adjusted relative risk of hypertension as compared with those in the lowest tertile; anger-in score was not associated with hypertension in women. In men and women, there were no associations between hypertension and either “anger-out” or tension scores. These findings generally support the hypothesis that—as is the case in Western populations—anger suppression and depression increase the risk of cardiovascular disease among Japanese.

## INTRODUCTION

In Japan, although the mortality rate of stroke has declined steadily from the 1960s to 2000,^1^ it is still higher than in the United States and Europe.^2^ However, the mortality rate of coronary heart disease (CHD) in Japan has been one-third to one-fifth that of the United States and Europe.^2^ Nevertheless, there is growing concern in Japan about a potential increase in the incidence and mortality of CHD, due to the Westernization of lifestyles, as typified by high-fat diets, widespread sedentary work patterns, and high levels of psychological stress.^3^

It has long been hypothesized that psychosocial factors such as depression, perceived stress, anger, and anxiety play a role in the development of cardiovascular disease (CVD). In Western populations, a number of prospective studies have found that these psychosocial characteristics are associated with an increased risk of cardiovascular morbidity and mortality.^4^^–^^7^ Few prospective studies, however, have addressed the association between psychological stress and CVD among Asian populations.^8^ Furthermore, it remains uncertain as to whether such psychosocial effects may be causal, and through which pathophysiological mechanisms they might be mediated.

The present study used data from the Circulatory Risk in Communities Study (CIRCS), a large population-based study of CVD and its predictors, to examine the associations of depression, anger, and anxiety with the incidence of CVD and its risk factors.

## OVERVIEW

### Study population

CIRCS is a population-based study of CVD incidence, risk factors, and trends within Japanese communities. Details of the study design and procedures have been published elsewhere.^3^^,^^9^^,^^10^ In brief, the subjects were Japanese men and women living in the northeastern rural community of Ikawa (total census population of 6206 in 1995), the southwestern rural community of Noichi (total census population of 15 828 in 1995), the central rural community of Kyowa (total census population of 17 322 in 1995), and a southwestern urban suburb—the Minamitakayasu district of Yao (total census population of 23 654 in 1995). Research teams from the Osaka Medical Center for Health Science and Promotion, the University of Tsukuba, Ehime University, and Osaka University have conducted annual cardiovascular risk surveys since 1963 in the districts of Yao City, Ikawa, and Noichi, and since 1981 in Kyowa. Informed consent was obtained from the community representatives to conduct an epidemiological study based on the guidelines of the Council for International Organizations of Medical Science. CIRCS was approved by the Ethics Committees of the Osaka Medical Center for Health Science and Promotion, University of Tsukuba, and Osaka University.

### Measurements of cardiovascular risk factors

Systolic and fifth-phase diastolic blood pressure were measured in each subject’s right arm by trained physicians using standard mercury sphygmomanometers. Serum total cholesterol was measured using the Liebermann-Burchard direct method between 1975 and 1986, and with an enzymatic method from 1986 onwards. All measurements were performed at the laboratory of the Osaka Medical Center for Health Science and Promotion, an international member of the US National Cholesterol Reference Method Laboratory Network.^11^ Body mass index (BMI) was calculated as weight (kg)/height (m)^2^. A 12-lead electrocardiogram (ECG) tracing was obtained in the supine position and coded with the Minnesota Code (second version) by trained physician-epidemiologists.

An interviewer obtained data on smoking status and weekly alcohol intake in units of “*go*” (a Japanese traditional unit of volume equal to 23 grams of ethanol), which was later converted to grams of ethanol per day. Those consuming more than 0.3 *go* per week were classified as current drinkers. Participants who smoked at least 1 cigarette per day were classified as current smokers.

### Endpoint determination

For all residents of the studied communities, CVD endpoints were ascertained from death certificates; national health insurance claims; reports of local physicians, public health nurses, and health volunteers; and/or annual cardiovascular risk surveys. To ascertain a diagnosis of CVD among living cases, medical histories and medical records from local clinics and hospitals were reviewed. In cases where the subjects had died, histories were obtained from their families, and by reviewing medical records.

The type of stroke (ie, intraparenchymal hemorrhage, subarachnoid hemorrhage, and ischemic stroke) was determined primarily by using CT/MRI findings.^12^ Stroke cases that were diagnosed clinically, but with no obvious radiological lesions on CT/MRI, were regarded as unclassified strokes. Stroke cases without CT/MRI films were classified using clinical criteria.

Definite myocardial infarction (MI) was defined as characteristic severe chest pain persisting at least 30 minutes with no definite nonischemic cause, accompanied by new, abnormal, and persistent Q or QS waves; and/or consistent changes in cardiac enzyme levels. If ECG and serum enzymes were nondiagnostic or unobtainable in patients with a clinical presentation that was characteristic of MI, a diagnosis of possible MI was made. Sudden cardiac death was defined as death within 1 hour of the onset of symptoms, a witnessed cardiac arrest, or abrupt collapse preceded by not more than 1 hour of symptoms. CHD was classified as definite MI, possible MI, or sudden cardiac death.

### Major findings

CIRCS has reported trends in the incidence of stroke, CHD, and cardiovascular risk factors in Japanese populations.^3^^,^^9^^,^^10^ The incidence of CHD among middle-aged urban-dwelling Japanese men increased between the periods from 1980 to 1987 and from 1996 to 2003, while no changes were seen in CHD incidence among urban-dwelling women or rural-dwelling men and women.^3^ CIRCS has also reported risk factors for stroke and CHD by using prospective analyses. Hypertension,^13^ smoking,^13^ high serum cholesterol,^13^ high serum triglycerides,^14^ high plasma fibrinogen,^15^ low serum HDL cholesterol,^16^ low alcohol consumption,^17^ and metabolic syndrome^18^ were all associated with increased CHD risk. Although hypertension and smoking are risk factors for stroke,^9^^,^^19^^,^^20^ serum total cholesterol level is inversely associated with the incidence of hemorrhagic stroke.^9^ In addition to these traditional risk factors, diabetes mellitus,^21^ major or minor ST-T abnormalities on ECG,^22^ increased carotid artery intima-media thickness,^23^ high serum homocysteine,^24^ high serum gamma-glutamyl transpeptidase (γ-GTP),^25^ and low intake of linoleic acid^26^ were reported to be risk factors for ischemic stroke in CIRCS. Furthermore, heavy drinking,^27^ low dietary intakes of saturated fat and animal protein,^28^ and high plasma fibrinogen were associated with an increased risk of intraparenchymal hemorrhage.^29^

## MEASUREMENT OF PSYCHOLOGICAL VARIABLES

### Depression

In October 1985, depressive symptoms were measured in 901 male and female residents of Kyowa aged 40 to 78 years by using the Zung Self-Rating Depression Scale (SDS).^30^ The original English scale was translated into Japanese, and the Japanese version has been well validated.^31^ Each of the 20 items on the Zung SDS is scored on a standard 4-point scale (1 to 4); the score range is thus 20 to 80. Individuals with a history of stroke (*n* = 14) or coronary heart disease (*n* = 8) were excluded, and data from 311 men and 568 women were used in the analyses.

### Anger expression

To measure anger expression, we used the Spielberger Anger Expression Scale,^32^ which is based on the frequency of reactions to anger-provoking situations. Anger expressed outwardly is regarded as “anger-out”, and anger held in or suppressed is classified as “anger-in.” We used 8 anger-out items, such as “express my anger”, “do things like slam doors”, and “argue with others”, and 8 anger-in items such as “keep things in”, “withdraw from people”, and “be secretly quite critical of others.” Using a 4-point scale, the subjects indicated the extent to which each statement described their general feelings or actions when they experienced anger or frustration. The scale was: 1 (“almost never”), 2 (“sometimes”), 3 (“often”), and 4 (“always”). Scores from the 8 items each for anger-out and anger-in were summed to produce a total anger expression score. Our previous report indicated that these measures are valid and reliable in Japanese populations.^33^

### Tension-anxiety

Tension-anxiety was measured using the Framingham Tension Scale.^34^ Seven tension items were used in the present study, including “troubled by feelings of tension, tightness, restlessness, or inability to relax” and “bothered by nervousness or shaking.” The subjects indicated their response on a 4-point scale as follows: 1 (“almost never”), 2 (“sometimes”), 3 (“often”), and 4 (“always”). Scores from the 7 items for tension were summed to produce a total tension score. In CIRCS, anger expression and tension scales were measured among 6292 men and women aged 30 to 74 years in Ikawa, Kyowa, Yao, and Noichi between 1995 and 1998.

## DEPRESSION AND CARDIOVASCULAR DISEASE

### Depression and incidence of coronary heart disease^35^

Among 879 men and women followed for 10.3 years, there were 21 incident cases of CHD. As compared with the lowest tertile of SDS scores, the age- and sex-adjusted hazard ratio of CHD for the highest tertile of SDS scores was 7.1 (95% confidence interval [CI], 2.0 to 25.7; Figure). This association was unchanged after further adjustment for BMI, systolic blood pressure, serum total cholesterol, alcohol intake, cigarette smoking, use of antihypertensive medication, and history of diabetes mellitus.

**Figure.**
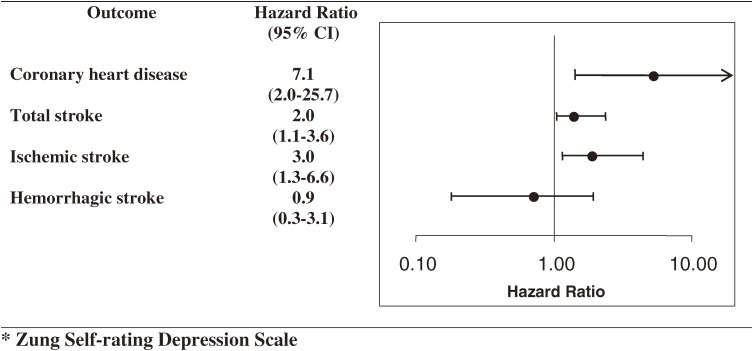
Age- and sex-adjusted hazard ratios (95% confidence interval) for coronary heart disease, total stroke, and stroke subtypes for the highest versus lowest tertiles of SDS* scores

### Depression and incidence of stroke^36^

There were 69 incident strokes during the 10.3-year period: 39 ischemic strokes, 20 hemorrhagic strokes (10 intracerebral and 10 subarachnoid hemorrhages), and 10 unclassified strokes. The age- and sex-adjusted prevalence of mild depression (SDS score ≥40) was 2 times higher among subjects with stroke than among those without stroke. As compared with participants in the lowest tertile of SDS scores, the age- and sex-adjusted hazard ratio of total stroke for those in the highest tertile of SDS scores was 2.0 (95% CI, 1.1 to 3.6; Figure). Although further adjustment for covariates reduced the relative risk to 1.9, this risk was still statistically significant. However, this excess risk was limited to ischemic stroke. The adjusted hazard ratios of ischemic and hemorrhagic strokes were 3.0 (1.3 to 6.6) and 0.9 (0.3 to 3.1), respectively.

## ANGER EXPRESSION, TENSION, AND BLOOD PRESSURE

### Cross-sectional associations^37^

There was an inverse association of anger-out expression with systolic and diastolic blood pressure levels in men. A 4-point (1 standard deviation) lower anger-out score was associated with a 1.6 mm Hg (95% CI, 0.6 to 2.6) increase in systolic blood pressure and a 0.6 mm Hg (−0.03 to 1.2) increase in diastolic pressure, after adjustment for age, BMI, alcohol intake, smoking status, and parental history of hypertension. The prevalence of hypertension also decreased with higher anger-out scores in men. The adjusted relative odds of hypertension for the lowest versus the highest tertiles of anger-out was 1.60 (1.19 to 2.15). The inverse association of anger-out with blood pressure was also observed among male workers who had no or few stress-coping behaviors.^38^ In women, the anger-out score was not associated with blood pressure. There was no association between blood pressure and anger-in or tension scores in either men or women.

### Longitudinal associations^35^

After excluding participants with baseline hypertension, annual surveys were used to follow the remaining 4970 men and women to determine the incidence of hypertension. A total of 3944 participants (1356 men and 2588 women, mean age 54 years) were re-examined in annual surveys and were included in the analyses. After an average follow up of 4 years, 451 participants (172 men and 279 women) developed hypertension during this period. Men with anger-in scores in the highest tertile had a 1.5-fold age-adjusted relative risk of hypertension, as compared with those with scores in the lowest tertile; the relative risk was 1.51 (95% CI, 1.05–2.17). This association was virtually unchanged after further adjustment for BMI, alcohol intake, and systolic blood pressure levels at baseline. In women, anger-in score was not associated with hypertension. In men and women, there was no relation between hypertension and either anger-out score or tension score.

In CIRCS, the anger-out score was inversely associated with hypertension in cross-sectional observation, while the anger-in score was positively associated with hypertension in longitudinal observation. Although we have no clear explanation for this difference, these findings support the hypothesis that anger suppression increases the risk of hypertension, which predisposes individuals to development of CVD. However, these findings must be replicated in other settings before any conclusions can be drawn.

## PATHOPHYSIOLOGICAL MECHANISMS

### Depression and cardiovascular disease

Depression can adversely affect a person’s quality of life by causing a deterioration in diet and physical activity. In a study of 7947 Japanese men and women, several factors (including sedentary lifestyle, insufficient sleep, and tendency toward irregular meal times) were associated with both perceived stress and depressive symptoms.^39^

In CIRCS, depressive symptoms were associated with risks for CHD and ischemic stroke, but not hemorrhagic stroke. It is possible that depressive symptoms increase the risk of CVD through increased platelet activity resulting from sympathoadrenal hyperactivity. As compared with healthy controls, depressed individuals have higher levels of platelet activation, as demonstrated by increased binding of monoclonal antibodies (ie, annexin V protein).^40^ Patients with depression and ischemic heart disease (IHD) also have higher plasma concentrations of platelet factor 4 and β-thromboglobulin than do IHD patients without depression or healthy control subjects.^41^ Furthermore, platelet secretion in response to collagen is significantly reduced among depressed patients after treatment with sertraline, an agent widely used for depression.^42^ These findings suggest that depression increases the risk of ischemic stroke via increased platelet aggregation.

Neurohormonal dysregulation, such as altered autonomic nervous system function, is one of several plausible mechanisms by which depression may predispose individuals to CVD. Increases in heart rate (HR), and diminished HR variability, are associated with higher incidences of CHD and cardiovascular death, and with rapid progression of coronary artery disease.^43^ In the Multi-Ethnic Study of Atherosclerosis (MESA), depressive symptom scores were positively associated with HR and inversely associated with HR variability.^44^ Interestingly, there was no association of trait anger or trait anxiety with HR and HR variability in MESA. It can be surmised from these findings that there are differences in the pathophysiological mechanisms of CVD with respect to depression, anger, and anxiety.

### Anger/hostility and cardiovascular disease

A number of prospective studies have reported associations of anger and hostility with incidences of CHD and stroke. Our longitudinal findings in CIRCS suggest that anger suppression increases the risk of CVD, at least in part through the development of hypertension. This is consistent with previous studies conducted in Western countries.^45^

Anger and hostility have also been associated with adverse lifestyle behaviors, such as excess alcohol consumption and smoking. Higher hostility scores were associated with higher values for BMI, alcohol intake, total energy intake, and proportion of current smoking.^46^ Furthermore, in the same study, hostility scores at baseline were inversely associated with serum levels of carotenoids, including α-carotene, β-carotene, β-cryptoxanthin, and zeaxanthin/lutein, at 7 years from baseline.^46^ Lower plasma levels of carotenoids (α-carotene, β-carotene, and lycopene) are positively related to ischemic stroke risk, as shown in the Physicians’ Health Study.^47^ Other putative mechanisms linking anger and hostility to the development of CVD include the hypothalamo-pituitary-adrenal (HPA) axis, platelet aggregation, blood pressure reactivity, metabolic syndrome, progression of carotid artery wall atherosclerosis, and neuroimmune modulation of inflammatory processes.

## FUTURE PROSPECTS

Further cross-sectional and longitudinal epidemiological studies are needed in CIRCS to elucidate the specific mechanisms responsible for the possible chronic effects of depression and anger on the incidence of CVD. Future prospective analyses should be conducted to examine associations of anger expression with CHD incidence and stroke using the abovementioned baseline data. Although previous studies have examined associations between negative psychological conditions and CVD, few have reported the effect of positive psychological conditions on CVD risk. A recent prospective study of 88 175 Japanese men and women showed that a lower perceived level of life enjoyment was associated with higher CVD risk and mortality among middle-aged men.^48^ For this reason, positive emotions and behaviors—like laughter, humor, and optimism—may reduce the risk of CVD among Japanese, although these associations need to be confirmed in further studies.

## CONCLUSIONS

In CIRCS, participants who reported a higher level of depressive symptoms had higher risks of CHD and ischemic stroke than did those who reported fewer such symptoms. No association was observed between depressive symptoms and hemorrhagic stroke. Men who suppressed their anger had an increased risk of hypertension; however, there was no association between anger expression and hypertension among women. These findings generally support the hypothesis that, as is the case in Western populations, anger suppression and depression increase the risk of CVD among Japanese.

## APPENDIX

### CIRCS collaborators

The Circulatory Risk in Communities Study (CIRCS) is a collaborative study managed by the Osaka Medical Center for Health Science and Promotion, University of Tsukuba, Osaka University, and Ehime University. The CIRCS investigators who contributed to this study are as follows: Masamitsu Konishi, Yoshinori Ishikawa, Akihiko Kitamura, Masahiko Kiyama, Masakazu Nakamura MD, Takeo Okada, Kenji Maeda, Masatoshi Ido, Masakazu Nakamura PhD, Mitsumasa Umesawa, Takashi Shimamoto, Minoru Iida, and Yoshio Komachi, Osaka Medical Center for Health Science and Promotion, Osaka; Shinichi Sato, Chiba Prefectural Institute of Public Health, Chiba; Tomonori Okamura, National Cardiovascular Center, Osaka; Yoshihiko Naito, Mukogawa Women’s University, Nishinomiya; Tomoko Sankai, Kazumasa Yamagishi, Kimiko Yokota, and Minako Tabata, University of Tsukuba, Tsukuba; Hiroyasu Iso, Tetsuya Ohira, Hironori Imano, Renzhe Cui, Satoyo Ikehara, Kotatsu Maruyama, Yoshimi Kubota, and Isao Muraki, Osaka University, Suita; Takeshi Tanigawa, Isao Saito, Katsutoshi Okada, and Susumu Sakurai, Ehime University, Tōon; Masayuki Yao, Ranryoen Hospital, Ibaraki; and Ai Ikeda and Hiroyuki Noda, Harvard School of Public Health, Boston, MA, USA.
